# Serum Activity of Liver Enzymes Is Associated With Higher Mortality in COVID-19: A Systematic Review and Meta-Analysis

**DOI:** 10.3389/fmed.2020.00431

**Published:** 2020-07-22

**Authors:** Umesha Boregowda, Mark M. Aloysius, Abhilash Perisetti, Mahesh Gajendran, Pardeep Bansal, Hemant Goyal

**Affiliations:** ^1^Department of Internal Medicine, Bassett Medical Center, Cooperstown, NY, United States; ^2^Department of Internal Medicine, The Wright Center for Graduate Medical Education, Scranton, PA, United States; ^3^Department of Gastroenterology and Hepatology, The University of Arkansas for Medical Sciences, Little Rock, AR, United States; ^4^Paul L. Foster School of Medicine, Texas Tech University Health Sciences Center El Paso, El Paso, TX, United States; ^5^Division of Gastroenterology, Moses Taylor Hospital and Reginal Hospital of Scranton, Scranton, PA, United States; ^6^The Wright Center for Graduate Medical Education, Scranton, PA, United States

**Keywords:** COVID-19, coronavirus, liver tests, liver chemistries, LFT, SARS-CoV-2

## Abstract

**Background:** Abnormal liver chemistries are common findings in patients with COVID-19. It is unclear whether abnormal liver chemistries can predict the severity of COVID-19. Therefore, we compared the serum liver chemistries such as hepatic transaminases, total bilirubin, albumin, and prothrombin time to evaluate whether they can predict severity and mortality in COVID-19.

**Methods:** An electronic search was performed on PubMed/Medline, EMBASE, and Google Scholar for studies comparing liver chemistries in severe and mild COVID-19. The literature search was performed using keywords “COVID-19,” “Liver,” Aspartate Aminotransferase (AST),” and “Alanine Aminotransferase (ALT),” “AST,” and “ALT,” in various combinations of “AND/OR” from December 1, 2019, till May 8, 2020. The pooled weighted mean difference (WMD) and 95% confidence interval (CI) were calculated for each component of liver chemistries.

**Results:** Twenty-two studies were eligible, with 3,256 patients (54.57% males). Seventeen studies compared liver chemistries for severe vs. mild COVID-19, whereas five studies compared liver chemistries in survival vs. non-survival groups. The pooled WMD of AST and ALT in severe vs. mild COVID-19 were 12.23 (95% CI; 8.07, 16.39; *p* < 0.01) and 8.07 (95% CI 2.55, 11.91; *p* < 0.01), respectively. The pooled WMD for AST in survivors vs. non-survivors analysis was 8.82 (*n* = 789; 95% CI; 2.27, 15.37; *p* < 0.01) and that of ALT was 4.70 (*n* = 340; 95% CI 0.04,9.35; *p* = 0.05).

**Conclusion:** Our meta-analysis shows that deranged liver chemistries may indicate severe COVID-19 and could also predict mortality. Larger studies are needed to evaluate the relationship between derangement in liver chemistries and mortality in COVID-19.

## Background

The coronavirus disease 2019 (COVID-19) started in December of 2019 as a cluster of pneumonia cases of unclear etiology in Wuhan city of Hubei province in China. Later, it was found to be caused by a coronavirus named severe acute respiratory syndrome coronavirus 2 (SARS-CoV-2). The disease rapidly spread throughout the world, becoming a pandemic, and as of May 11, 2020, there were > 4 million cases worldwide with >278,000 deaths (1). There have been >1.3 million cases with >79,000 deaths in the United States (US) as per the Center for Disease Control and Prevention (CDC) (2). It is postulated that the virus enters the human body by binding to the angiotensin-converting enzyme receptor-2 (ACE-2) with the help of spike protein on its cell membrane (3). Common clinical manifestations of COVID-19 include fever, cough, shortness of breath, and fatigue. However, gastrointestinal (GI) manifestations such as nausea, vomiting, anorexia, diarrhea, and abdominal pain are also commonly reported now (4, 5). Recently, new-onset loss of taste or smell has also gained attention as important symptoms of COVID-19 (6, 7). The reason for these extrapulmonary symptoms could be the presence of ACE-2 receptors of SARS-CoV-2, in the gastrointestinal tract, liver, and pancreas.

About 80% of the patients with COVID-19 have mild disease. Patients with severe disease have a poor prognosis and have a higher risk of mortality from respiratory failure (8). COVID-19 is characterized by several laboratory abnormalities such as elevated lactate dehydrogenase (LDH), D-dimer, cardiac troponin I, procalcitonin, ferritin, transaminases, bilirubin, and lower lymphocyte count (9). Hence, it is crucial to understand how these laboratory abnormalities could be used to prognosticate the disease for clinical outcomes. The incidence of liver injury has been reported in the range of 16–53% by several retrospective studies (10, 11). It has also been noted that patients with severe COVID-19 have higher odds of abnormal liver chemistries when compared to patients with mild COVID-19 (12–14). However, their value as prognostic markers is not clear. In a study by Wang et al., patients requiring admission to intensive care unit (ICU) had alanine aminotransferase (ALT) elevated at 1.5 times of normal levels, aspartate aminotransferase (AST) was elevated at 1.8 times the normal levels, and total bilirubin (TB) 1.2 times the normal levels (14). Similarly, in a study by Huang et al., abnormal ALT (1.8-fold increase), TB (1.3-fold increase), and albumin (20% decrease) were significant predictors of ICU admission (13).

There is a paucity of reliable data on the prognostication ability of deranged liver chemistries. Therefore, we performed a systematic review and meta-analysis by combining relevant studies to evaluate the patterns of abnormal liver chemistry and their use as prognostic markers for the severity and mortality related to COVID-19. The aim was to analyze the pooled weighted mean difference (WMD) of liver chemistries between severe and mild COVID-19 as well as survivors and non-survivors with COVID 19 (12, 13).

## Methods

This meta-analysis was performed as per the Preferred Reporting Items For Systematic Review And Meta-Analysis Statement' (PRISMA) (15).

### Population, Interventions, Comparators, Outcomes, and Study Designs (PICOS)

Population: Patients who were admitted to hospitals with COVID 19.
Intervention: To evaluate the significance of elevated serum liver enzymes activity in COVID 19
Comparators: Serum liver enzyme activity in mild and severe COVID 19 as well as survivors and non-survivors with COVID 19
Outcomes: Serum AST, ALT, Albumin, PT, and total bilirubin in COVID 19 patients
Study designs: A systematic review and meta-analysis.

### Search Strategy

We performed an electronic search on online databases PUBMED/Medline, EMBASE, and Google Scholar from Dec 2019 to May 8, 2020, for articles published on abnormal liver chemistries, severity, and mortality related to COVID-19. Studies that reported AST, ALT, TB, prothrombin time (PT), and albumin as “median and range” were included. The search for literature was performed using keywords “COVID-19 AND Liver,” COVID AND AST,” “COVID AND ALT,” “COVID AND Aspartate Aminotransferase,” and “COVID AND Alanine Aminotransferase.” Further attempts were made to identify articles by manually searching the references used in these articles.

### Study Selection Criteria

Studies that met the following inclusion criteria were included in the meta-analysis; studies that compared liver chemistries in severe and mild COVID-19 patients or studies that compared serum liver enyme activity in survivors and non-survivors. Studies using the data only from the confirmed COVID-19 hospitalized patients were included. The definition of severe COVID-19 included either respiratory rate >30/min, hypoxemia (oxygen saturation <93% on room air at rest), or the ratio of the partial pressure of arterial oxygen to fraction of inspired oxygen (FiO_2_) <300 mmHg or a combination of these findings. Patients were considered critically ill if they required admission to the ICU for mechanical ventilation or vasoactive agents for shock or due to organ failure. The articles which were in the format of case reports, reviews, non-human studies, abstract only, and editorials were excluded.

### Data Extraction

Two investigators (UB and MM) independently reviewed the included studies and tabulated the data into a standardized data collection sheet. A third author (HG) reviewed the studies independently whenever a lack of consensus on eligibility was determined and made the final decision regarding data collection. The data extracted from the included studies were the author's name, year of publication, study location, type of study, the total number of patients, participants' gender, the average age of the subjects, AST, ALT, PT, TB, and albumin values in (median and range or mean and SD) for severe vs. mild COVID-19 patients, as well as survivors vs. non-survivors.

### Quality Assessment

Quality assessment of each study included in the meta-analysis was performed using the Cochrane risk of bias tool. The study quality was assessed based on the selection of study subjects, blinding of the subjects, outcome assessment, completeness of data, and reporting of the outcomes.

### Outcomes

The primary endpoints were pooled WMD of aminotransferases (AST and ALT) between severe and mild COVID-19, as well as in the survivors and non-survivors. The secondary endpoints were pooled WMD of TB, PT, and albumin in “mild vs. severe COVID-19” and “survivors and non-survivors.”

### Statistical Analysis

The pooled estimates of WMD with a 95% confidence interval (CI) for the outcomes of interest were synthesized through meta-analysis using the DerSimonian-Laird random-effects model. The mean difference and 95% CI were extrapolated from the median and range for each study as per Hozo et al. (16). The heterogeneity among the included studies was estimated by calculating the inconsistency index (*I*^2^). Heterogeneity was classified as low, moderate, and substantial heterogeneity if the scores were 25, 50, and 75%, respectively. Publication bias analysis was performed using the funnel plot. Sensitivity analyses were performed by the exclusion of individual studies separately, and their effect was noted to be significant if this changed the outcome of the pooled data, which was reported accordingly. RevMan software (Review Manager Version 5.3; The Nordic Cochrane Center, Copenhagen, Denmark, The Cochrane Collaboration 2015) was used for statistical evaluation.

## Results

**Figure 1:**
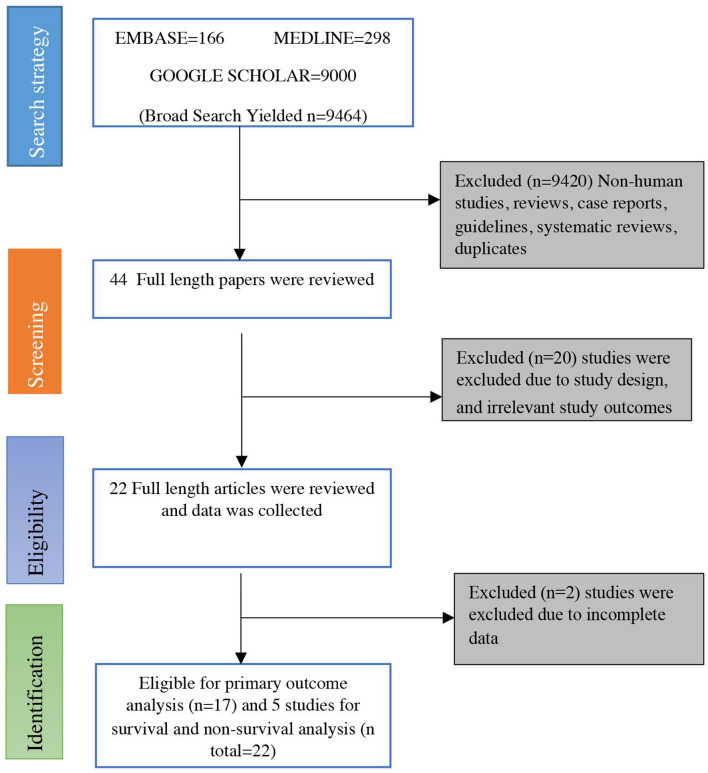
Flow chart of selecting eligible studies for the meta-analysis.

A search strategy using three search engines (PubMed/Medline, EMBASE, and Google Scholar) on April 8, 2020, using the keywords mentioned above yielded 9,499 articles initially. Of these, 9,455 were excluded because they were in the formats of case reports, guidelines, reviews, non-human studies, and/or duplicates. The remaining 44 studies were screened, of which 20 were rejected due to improper study design or irrelevant outcomes. Twenty four studies were deemed eligible initially, but two were further excluded due to incomplete data (12, 17). Finally, 22 studies that met the inclusion criteria were included in the meta-analysis (13, 14, 18–37). A flow diagram for the process of literature search and study selection is shown in Figure 1.

Twenty-two studies reported AST or ALT with a total of 3,256 patients, of which 1,777 were males (54.57%). Seventeen studies compared liver chemistry abnormalities in the mild and severe COVID-19 groups (13, 18–32, 34, 36), and the remaining five studies compared liver chemistry abnormalities among the survivors and non-survivors (33–35, 37). A list of included studies and their details are provided in Table 1.

**Table 1:** List of studies included in the meta-analysis.

**References**	**Year**	**Country**	**Type of study**	**Total *n***	**Males total**	**Comparison**	**Severe COVID-19/died *n***	**Males Severe COVID-19**	**Age in median (range) or Mean *(SD)***	**Mild COVID-19/survivors *n***	**Males in mild COVID-19**	**Age in median (range) or Mean *(SD)***
Cai et al. (18)*	2020	China	Retrospective study	318	151	Mild vs. severe	85	NA	NA	233	NA	NA
Wang et al. (14)	2020	China	Retrospective study	138	75	Mild vs. severe	36	22	66 (57–78)	102	53	51 (37–62)
Deng et al. (33)	2020	China	Retrospective study	91	86	Survivors vs. non-survivors	109	73	69 (62–74)	116	51	40 (33–57)
Fu et al. (20)*	2020	China	Retrospective study	299	159	Mild vs. severe	88	NA	NA	211	NA	NA
Huang et al. (13)	2020	China	Prospective study	41	86	Mild vs. severe	13	11	49 (41–61)	28	19	49 (41–57·5)
Wang et al. (38)	2020	China	Retrospective study	339	86	Survivors vs. non-survivors	65	50	76 (70–83)	274	127	68 (64–74)
Liu et al. (21)	2020	China	Retrospective study	30	10	Mild vs. severe	4	NA	NA	26	NA	NA
Mo et al. (22)	2020	China	Retrospective study	155	86	Mild vs. severe	85	55	61 (51–70)	70	31	46 (35–56)
Qian et al. (23)	2020	China	Retrospective study	91	37	Mild vs. severe	9	NA	66 (54–80)	82	NA	49 (35.3–56)
Qu et al. (24)	2020	China	Retrospective study	30	16	Mild vs. severe	3	NA	60 ± 5.29	27	NA	49.44 ± 14.86
Ruan et al. (35)	2020	China	Retrospective study	150	102	Survivors vs. non-survivors	68	49	67 (15–81)	82	53	50 (44–81)
Tianxin et al. (25)	2020	China	Retrospective study	49	33	Mild vs. severe	9	8	53.0 ± 14	40	25	40.6 ± 14.3
Liu et al. (26)	2020	China	Retrospective study	78	86	Mild vs. severe	11	7	66 (51–70)	67	32	37 (32, 41)
Wan et al. (27)	2020	China	Prospective study	135	86	Mild vs. severe	40	21	56 (52–73)	95	52	44 (33!49)
Wu et al. (28)	2020	China	Retrospective study	201	128	Mild vs. severe	84	60	58.5 (50–69)	117	68	48 (40–54)
Wu et al. (28) subgroup	2020	China	Retrospective study	84	60	Survivors vs. non-survivors	44	29	68.5 (59.3–75)	40	31	50 (40.3–56.8)
Xu et al. (32)	2020	China	Retrospective study	62	35	Mild (<10 days) vs. severe (>10 days)	33	19	45 (37–54)	29	16	39 (31–50)
Yang et al. (36)	2020	China	Retrospective study	52	86	Survivors vs. non-survivors	32	21	64.6 (11.2)	20	14	51.9 (12.9)
Yudong et al. (29)	2020	China	Retrospective study	112	86	Mild vs. severe	16	9	57.5 (54–63)	96	44	62 (55–67.5)
Yun et al. (30)	2020	China	Retrospective study	292	154	Mild vs. severe	21	19	65.5 ± 15.7	271	135	48.7 ± 15.7
Wang et al. (14)	2020	China	Retrospective study	69	32	Mild vs. severe	14	7	70.5 (62–77)	55	25	37 (32–51)
Zhang et al. (31)	2020	China	Retrospective study	115	49	Mild vs. severe	31	20	64.58 ± 13.26	84	29	43.96 ± 14.84
Zhou et al. (37)	2020	China	Retrospective study	191	86	Survivors vs. non-survivors	54	38	69 (63–76)	137	81	52 (45–58)

COVID-19- Coronavirus 2019, NA, Not available. *Study demographics in percentage used to calculate gender number.

### Liver Chemistry in Mild vs. Severe COVID-19

**Figure 2:**
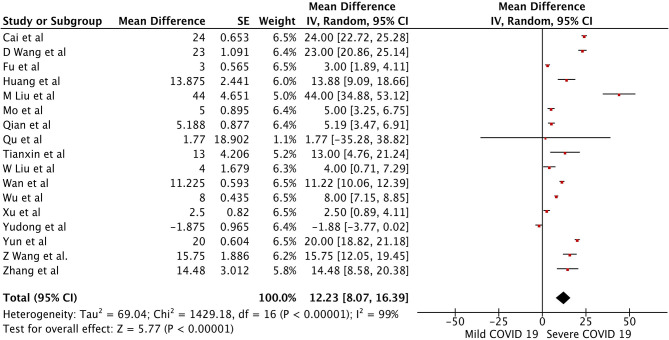
Forest plot for the pooled weighted mean difference of AST in mild vs. severe COVID-19.

**Figure 3:**
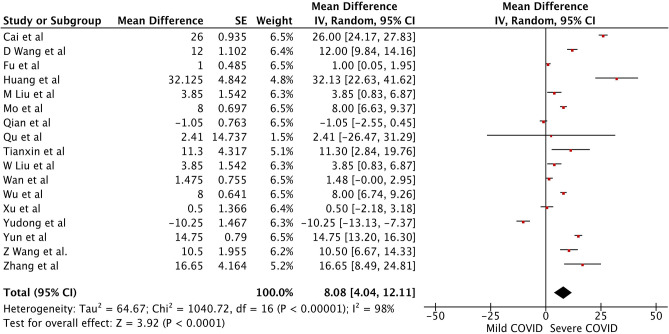
Forest plot for the pooled weighted mean difference of ALT in mild vs. severe COVID-19.

There were a total of 2215 patients in the severe vs. mild COVID-19 groups from 17 studies with 1160 males (52.37%). Two of the seventeen studies were prospective cohort studies (13, 37), and the remaining 15 were retrospective studies. There were a total of 582 (26.26%) patients with severe disease and 1,633 patients (73.4%) with mild COVID-19. The pooled WMD of AST was 12.23 (*n* = 2,215; 95% CI 8.07, 16.39; *p* < 0.01) (Figure 2). The pooled WMD of ALT was 8.07 (*n* = 2,215; 95% CI 2.55, 11.91; *p* < 0.01) (Figure 3). Sensitivity analysis was performed to evaluate the effect of individual studies on the pooled estimates, and no significant difference was observed.

**Figure 4:**
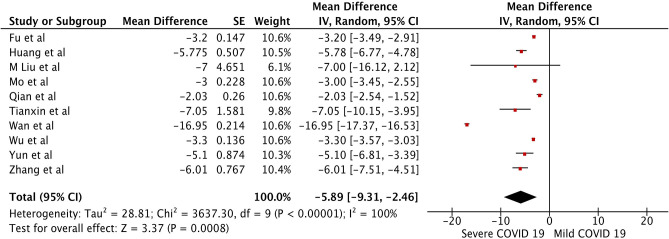
Forest plot for the pooled weighted mean difference of Albumin in mild vs. severe COVID-19.

The pooled WMD of albumin in mild vs. severe COVID-19 was −5.89 (*n* = 1,408; 95% CI −9.31, −2.46; *p* < 0.01) (Figure 4), indicating low albumin may be associated with severe COVID-19. The pooled WMD for PT, and TB, in the mild vs. severe COVID-19 analysis were 0.66 (*n* = 627; 95% CI 0.43,0.89; *p* < 0.01), and 2.32 (*n* = 1,408; 95% CI 1.54, 3.10; *p* < 0.01), respectively. However, there was significant heterogeneity among the included studies (*I*^2^ = 99%). Pooled estimates are listed in Table 2. The forest plots for PT and TB are shown in the Supplementary Figures 1, 2, respectively.

**Table 2:** Results for the primary and secondary outcomes.

**Liver function**	**Patients (*n*)/number of studies**	**WMD (95% CI)**	***I*^**2**^**	***P*-value**
**Mild vs. Severe COVID-19**				
Aspartate aminotransferase, U/L	2,215 (17)	12.23 [8.07, 16.39]	99%	<0.0001
Alanine aminotransferase, U/L	2,215 (17)	8.08 [4.04, 12.11]	98%	<0.0001
Albumin, g/L	1,408 (10)	−5.89 [−9.31, −2.46]	100%	0.0008
Total Bilirubin g/L	1,408 (9)	2.32 [1.54, 3.10]	94%	<0.00001
Prothrombin time in seconds	627 (5)	0.66 [0.43, 0.89]	89%	<0.00001
**Survivor vs. Non-survivors in COVID-19**				
Aspartate aminotransferase, U/L	174 (4)	8.82 [2.27, 15.37]	98%	0.008
Alanine aminotransferase, U/L	755 (5)	4.70 [0.04, 9.35]	97%	0.05
Albumin in g/L	426 (5)	−3.69 [−4.43, −2.95]	95%	<0.00001
Total Bilirubin g/L	4.04 [1.90, 6.18]	4.04 [1.90, 6.18]	52%	0.0002
Prothrombin time in seconds	582 (4)	0.71 [0.29, 1.13]	96%	0.001

*WMD, Weighted mean difference; CI, Confidence interval; I^2^, inconsistency index; COVID-19, Coronavirus disease 2019*.

### Liver Chemistry in Survival vs. Non-survival Groups With COVID-19

There were a total of 1,041 patients in the survival and non-survival groups, with 617 (59.26%) males from five studies (28, 33, 35, 37, 38). Results of a subgroup analysis from Wu et al. that compared survival and non-survival groups were also included in this analysis (28). The pooled WMD for AST in survivors vs. non-survivors analysis was 8.82 (*n* = 798; 95% CI; 2.27, 15.37; *p* < 0.01) and pooled WMD for ALT was 4.70 (*n* = 340; 95% CI 0.04,9.35; *p* = 0.05). Liver chemistries were significantly elevated in the non-survival group when compared to the survival group suggesting increased mortality in patients with elevated liver chemistries. The forest plot analysis of AST and ALT in survivor vs. non-survivor groups are depicted in the Supplementary Figures 3, 4, respectively. The WMD of albumin, PT, and TB in survivor vs. non-survivor groups were −3.69 (*n* = 425; 95% CI −4.43, −2.95; *p* < 0.01), 0.71 (*n* = 195; 95% CI 0.29, 0.97; *p* < 0.01), and 4.04 (*n* = 144; 95% CI 1.90, 6.18; *p* < 0.01), respectively. The forest plot analysis for albumin, PT, and TBili are shown in Supplementary Figures 5–7, respectively.

#### Publication Bias

Publication bias was assessed using a funnel plot analysis. Supplementary Figure 8 shows the clustering of studies toward the median and top of the funnel plot suggesting no significant publication bias in the studies included for the primary outcomes. However, a significant number of studies were outside the funnel likely due to the non-uniformity in study designs. Egger's *p*-value was insignificant (*p* = 0.759), indicating these outliers did not contribute to significant publication bias.

### Quality Assessment

Quality assessment of included studies in the meta-analysis was performed using the Cochrane risk of bias tool. The quality assessment of each included study is shown in Supplementary Table 1.

## Discussion

This meta-analysis shows that patients with severe COVID-19 have a significantly elevated AST, ALT, PT, and T. Bili when compared with mild COVID-19 patients. The pooled analysis also showed that non-survival groups had significantly elevated serum liver enzymes (AST and ALT) activity and PT when compared to survival groups. Pooled estimates also indicate severe COVID-19 and non-survival groups have low serum albumin level albumin levels. It is also observed that the mean age among the severe disease and non-survival groups were higher when compared to mild disease and survival group suggesting poorer outcomes with increasing age. Therefore, elevated liver enzymes, PT, low albumin levels advanced age should alert the care providers about possible deterioration and need for mechanical ventilation as well as increased mortality in these patients. Close monitoring and timely interventions can improve morbidity and mortality in these patients.

Based on the current literature, the prevalence of abnormal liver chemistries in COVID-19 is noted in the range of 16–53% by several studies (10, 11, 39). It is essential to recognize that patients with significantly elevated liver chemistries may experience severe disease, and therefore, are likely to be admitted to ICU when compared to patients who do not have liver chemistry abnormalities. Previous studies have shown that inflammatory markers such as C reactive protein (CRP), LDH, ferritin, interleukin-6, and lymphopenia can predict severity of COVID-19 (40, 41). Our meta-analysis discloses that serum activity of aminotransferase is not only associated with the severe COVID-19 but is also associated with higher mortality in this population. Liver injury in COVID-19 was reported by Chen et al., with nearly 43.4% of the patients from 99 cases developed elevation of transaminases (42). Although elevated liver chemistries are commonly noted in COVID-19 patients, the key area of interest is whether they could predict outcomes. Cai et al. reported abnormal liver tests in 76% (318/417) of patients (18). They reported that hepatocellular and mixed pattern of liver enzyme elevation is associated with higher odds of progression to severe disease (18). Few studies have reported longer length of stay with the elevation of any liver chemistries (43, 44), but it is unclear if liver dysfunction directly correlates with outcomes in COVID-19 patients due to lack of adjustment for potential confounders such as inflammatory markers (ferritin, lactate dehydrogenase, high white cell count). Extrahepatic sources such as muscle injury can also cause elevated AST and ALT (45). Therefore, it is paramount to differentiate AST and ALT elevation due to muscle injury from hepatic injury due to COVID 19. Gamma glutamyl transpeptidase (GGT) is highly specific to hepatic injury and can aide to differentiating the liver injury from muscle injury (46).

Though the exact mechanism of liver injury noted in COVID-19 is unclear, multiple hypotheses such as direct viral cytotoxicity through ACE-2, drug-induced liver injury, immune-mediated damage, and passive congestion have been proposed. The SARS-CoV-2 virus enters the human cells via its functional receptor- ACE-2. The concentration of this receptor is significantly higher in the biliary epithelium compared to hepatocytes. However, transaminitis suggestive of hepatocellular injury is observed more often than the cholestatic pattern with abnormal bilirubin levels (12). The exact cause for this variation is not known at this time and further studies are need to explore the possible mechanisms.

The SARS-CoV-2 virus incites an immune response causing CD4+T cell-dependent activation of B lymphocytes to cause increased antibody production. CD+8 T cells also play a significant role in clearing the virus, which is also found in the liver on autopsy (47). The autopsy of the patients who died from COVID-19 revealed mid-lobular and portal activity associated with microvascular steatosis (48). Other features of pathological liver changes on autopsy include, hepatomegaly with dark red hepatocyte degeneration, lobular focal necrosis with neutrophils, monocytes, and lymphocyte infiltration in the portal area. The congestion of the hepatic sinuses with micro-thrombosis have also been noted (49). An increased level of monocyte chemoattractant protein 1 (MCP-1) observed in COVID-19 can also exacerbate steatohepatitis (13, 50). The upregulation of ACE2 receptors due to a compensatory proliferation of hepatocytes may play a role in liver injury (51). Another potential cause of liver injury in COVID-19 is direct virus-mediated injury, which is not well-understood so far. Most patients with COVID are treated with acetaminophen for fever, which can cause liver injury. Antiviral medications such as oseltamivir, lopinavir/ritonavir may also cause liver injury, and caution is required while using these drugs (52). Patients who are on hepatotoxic medications for other medical conditions (e.g., HIV medications, anti-tubercular medications, and chemotherapy agents) are at increased risk of developing a liver injury. Therefore, non-hepatotoxic agents should be considered in these patients for the treatment of COVID 19. It is not clear whether the presence of chronic liver disease in COVID-19 patients would affect the severity of the disease or mortality. Also, hypoxia associated with severe COVID-19 due to acute respiratory distress syndrome can also cause liver damage (53). Previous animal studies suggest hypoxia-induced vascular endothelial growth factor (VEGF) and collagen I expressions are associated with angiogenesis and fibrogenesis (54).

Elevated hepatic transaminases, PT, and low albumin levels indicate that COVID 19 affects both catbolic and anabolic activities of liver through hepatocyte injury. Hepatocyte dysfunction can lead to poor response to stress and infections due to decreased protein synthesis that are essential in fighting infections. This can lead to an immune suppression status making these patients vulnerable to secondary bacterial infections. Secondary bacterial infections can increase the morbidity and mortality in Severe COVID 19 patients.

## Strengths and Limitations

This meta-analysis highlights an important clinical manifestation in COVID 19, that is elevated liver enzymes may indicate severe COVID 19, and higher mortality. The data is valuable information to clinical providers for prognostication, guiding therapy and management of COVID 19 patients. Our study has some limitations that should be considered. The studies included in our meta-analysis are observational studies with significant heterogeneity. The majority of the studies did not report whether the patients included were consecutive patients, which increases the risk of selection bias. There was inconsistency in reporting the presence of chronic liver disease in these studies, which limits the ability to determine if the abnormalities in liver chemistries are acute or chronic. Some studies included in the mild and severe COVID-19 analysis had patients who were still hospitalized at the time of preparing the study results, which could have affected the outcomes. Also, most of these studies are from Wuhan, China, and therefore, results may not be generalizable. Lastly, most studies did not report the stratification of liver chemistry abnormalities. Therefore, we could not determine the cut off limit for liver chemistries that is clinically relevant to predict outcomes. Future studies should include data from a wider population in order to assess the validity of our findings.

Our meta-analysis has the most comprehensive review of parameters of liver tests performed for almost all the COVID-19 patients at the time of admission. Future studies evaluating liver dysfunction in COVID-19 patients should consider stratifying the severity of liver injury based on transaminase levels, the severity of COVID-19, the effect of COVID-19 on chronic liver disease, morbidity, and mortality. More extensive multicenter studies with sufficient follow up are needed to evaluate the mechanism of liver injury, its relation to severity and mortality due to COVID-19, and estimate the drug-induced liver injury in these patients. The role of COVID-19 liver injury in patients with chronic viral hepatitis (Hepatitis B and C), liver cirrhosis, and non-alcoholic fatty liver disease also needs to be evaluated.

In conclusion, our study shows that increased aminotransferase activity, PT, and low albumin levels in COVID-19 patients are associated with severe disease and higher mortality. Clinical providers caring for COVID 19 patients should recognize the presence of elevated liver enzymes that can indicate severe COVID 19 and adjust their treatment strategies accordingly. Close monitoring and follow ups may be the key in recognizing the deterioration of clinical conditions in these patients. Such adjustments to practice have the potential to avoid the delay in care and ultimately reduce the overall morbidity and improve survival among severe COVID 19 patients due to timely intervetions.

## Data Availability Statement

The original contributions presented in the study are included in the article/Supplementary Material, further inquiries can be directed to the corresponding author.

## Author Contributions

HG: conception and design. UB, MA, and HG: literature search. UB and MG: first draft. All authors: critical revision and editing and final approval.

## Conflict of Interest

The authors declare that the research was conducted in the absence of any commercial or financial relationships that could be construed as a potential conflict of interest.

## Supplementary Material

Supplementary Figure 1 Forest plot for the pooled weighted mean difference of Prothrombin time (PT).

Supplementary Figure 2 Forest plot for the pooled weighted mean difference of Total Bilirubin (TB).

Supplementary Figure 3 Forest plot for the pooled weighted mean difference of AST in survivor vs. non-survivors in COVID-19.

Supplementary Figure 4 Forest plot for the pooled weighted mean difference of ALT in survivors vs. non-survivors in COVID-19.

Supplementary Figure 5 Forest plot for the pooled weighted mean difference of Albumin in survivors vs. non-survivors.

Supplementary Figure 6 Forest plot the for the pooled weighted mean difference of prothrombin time (PT) in survivors vs. non-survivors in COVID-19.

Supplementary Figure 7 Forest plot for the pooled weighted mean difference of Total Bilirubin (TB) in survivors vs. non-survivors in COVID-19.

Supplementary Figure 8 Funnel plot for the pooled weighted mean difference of Total Bilirubin (TB) in mild vs. severe COVID-19.

Supplementary Table 1 The quality assessment of each included study performed using the Cochrane risk of bias tool.
