# Supplementary material for: Serum Activity of Liver Enzymes Is Associated With Higher Mortality in COVID-19: A Systematic Review and Meta-Analysis
Source: Front Med (Lausanne). 2020 Jul 22;7:431. doi: 10.3389/fmed.2020.00431 (PMC7387424; doi:10.3389/fmed.2020.00431)
Supplement: Supplementary Table 1 — The quality assessment of each included study performed using the Cochrane risk of bias tool. [file Data_Sheet_1.pdf]

Supplementary Table 1: The quality assessment of each included study performed using the Cochrane risk of bias tool.

	Random sequence generation (selection bias)	Allocation concealment (selection bias)	Blinding of participants and personnel (performance bias)	Blinding of outcome assessment (detection bias)	Incomplete outcome data (attrition bias)	Selective reporting (reporting bias)	Other bias
Cai et al	?	+	+	+	+	+	+
Deng et al	?	+	+	+	+	+	
D Wang et al	+	+	+	+	+	+	+
Fu et al	?	+	+	+	+	+	+
Huang et al	+	+	+	+	+	+	+
L Wang et al	+	+	+	+	+	+	+
M Liu et al	−	+	+	+	+	+	+
Mo et al	+	+	+	+	+	+	+
Qian et al	?	+	+	+	+	+	+
Qu et al	−	+	+	+	+	+	+
Ruan et al	?	+	+	+	+	+	+
Tianxin et al	?	+	+	+	+	+	+
Wan et al	?	+	+	+	+	+	+
W Liu et al	+	+	+	+	+	+	+
Wu et al	+	+	+	+	+	+	+
Xu et al	?	+	+	+	+	+	+
Yang et al	−	+	+	+	+	+	+
Yudong et al	+	+	+	+	+	+	+
Yun et al	?	+	+	+	+	+	+
Zhang et al	+	+	+	+	+	+	+
Zhou et al	+	+	+	+	+	+	+
Z Wang et al.	+	+	+	+	+	+	+
